# Environmental Maternal Effects Mediate the Resistance of Maritime Pine to Biotic Stress

**DOI:** 10.1371/journal.pone.0070148

**Published:** 2013-07-26

**Authors:** María Vivas, Rafael Zas, Luis Sampedro, Alejandro Solla

**Affiliations:** 1 Ingeniería Forestal y del Medio Natural, Universidad de Extremadura, Plasencia, Cáceres, Spain; 2 Misión Biológica de Galicia (MBG-CSIC), Pontevedra, Galicia, Spain; National Taiwan University, Taiwan

## Abstract

The resistance to abiotic stress is increasingly recognised as being impacted by maternal effects, given that environmental conditions experienced by parent (mother) trees affect stress tolerance in offspring. We hypothesised that abiotic environmental maternal effects may also mediate the resistance of trees to biotic stress. The influence of maternal environment and maternal genotype and the interaction of these two factors on early resistance of *Pinus pinaster* half-sibs to the *Fusarium circinatum* pathogen was studied using 10 mother genotypes clonally replicated in two contrasting environments. Necrosis length of infected seedlings was 16% shorter in seedlings grown from favourable maternal environment seeds than in seedlings grown from unfavourable maternal environment seeds. Damage caused by *F. circinatum* was mediated by maternal environment and maternal genotype, but not by seed mass. Mechanisms unrelated to seed provisioning, perhaps of epigenetic nature, were probably involved in the transgenerational plasticity of *P. pinaster*, mediating its resistance to biotic stress. Our findings suggest that the transgenerational resistance of pines due to an abiotic stress may interact with the defensive response of pines to a biotic stress.

## Introduction

A plant’s phenotype may depend not only on its genotype and the environmental conditions where it grows, as conventionally thought, but can also be determined by the environment experienced by the parents (mainly the mother) [1]. Transgenerational plastic responses to the maternal environment are transmitted to the offspring phenotype without any change in the DNA sequence [2]. As an important source of phenotypic variation, maternal environmental effects can influence the evolutionary process and population dynamics of many plant species [3], [4]. Increasing evidence indicates that transgenerational plasticity could be adaptive, enhancing offspring fitness under environments similar to the maternal environment [5]. Although much less studied, maternal environmental effects could also be potentially exploited to improve the performance of man-made plantations by exposing mother plants to appropriate environmental cues [6], [7].

The maternal environment is known to influence many traits, e.g. seed traits [8], germination [2], and seedling performance [9] in many different plant species, including long-lived plants such as conifers [7], [10]–[13]. One of the better known examples of transgenerational plasticity in conifers is the epigenetic memory reported for Norway spruce, in which the temperature and photoperiod experienced by the mother tree during embryo development modulated offspring tolerance to frost through growth phenology adjustments (reviewed in [7]). The effect is long lasting (up to 20 years), quantitatively important, and has been recognised as an important mechanism of rapid adaptation to environmental changes [14]. Similar transgenerational responses to climate cues have been reported in several other conifer species (see references in [7]).

Transgenerational responses to maternal environments are not restricted to abiotic cues. Biotic stresses are also known drivers of transgenerational phenotypic changes (reviewed by [15]). For example, maternal wild radish plants exposed to caterpillar herbivore damage produce more resistant offspring seedlings [16], and Arabidopsis transgenerational resistance to chewing herbivores could prime progeny plants for more enhanced resistance [17] than offspring from unthreatened parents. Most studies of transgenerational induction of defences to pests and pathogens in plants have focused on short-lived annuals, and it remains largely unknown whether this type of transgenerational plasticity also occurs in long-lived forest trees [15].

Environmental maternal effects can be transmitted to the next generation through diverse mechanisms with varied ecological and evolutionary implications [4]. On the one hand, the resources that mother plants allocate to seeds are environmentally dependent, and the amount and quality of the resources stored within seeds can greatly affect germination and early development of plants [12], [18], [19]. Seed provisioning is, therefore, an important transmission vehicle of environmental maternal effects [4] whose influence is usually restricted to one generation and normally diminishes with seedling age [20]. On the other hand, epigenetic mechanisms; i.e., a set of molecular processes that modulate the phenotype by modifying gene expression, contribute to transmit heritable plastic responses to environmental cues [21]. Transgenerational epigenetic changes include DNA methylation, histone modification and small RNA interference [15], [20], and may persist throughout the life cycle and even across multiple generations [22], [23]. Quantification of the relative contribution of resource-dependent and resource-independent mechanisms in the transmission of specific transgenerational plastic responses will help to understand the ecology and evolution of natural populations [9], [24] and could also be crucial in applied fields of science such as conservation biology and pest management.

Abiotic stressors in the maternal environment that elicit transgenerational plasticity and affect the performance of progeny when challenging environmental harshness have been widely reported in recent decades [4]. Evidence similarly shows that biotic stresses exerted on mother plants by herbivores or pathogens can induce transgenerational defences in progeny [15]. However, little is known about whether abiotic stress in the maternal environment can also be associated with transgenerational defensive plasticity. This study aims to elucidate whether maternal environments strongly differing in abiotic characteristics have any influence on early resistance of *Pinus pinaster* seedlings to the fungal pathogen *Fusarium circinatum* and the extent to which differences in seedling performance and resistance between maternal environments are mediated by seed provisioning.

*Pinus pinaster* is a native conifer of the Western Mediterranean basin with great importance to the economy of this area. The virulent *F. circinatum* fungus causes pitch canker disease in pines. This invasive forest pathogen is native to North America but has been recorded in various countries outside its natural range [25] and has greatly impacted most *Pinus* plantations worldwide [26]. In Spain, it was first isolated in nurseries and forest plantations of *P. radiata* and *P. pinaster* [27] and is now apparently well established in the north, representing the main threat to *Pinus* in this area [28], [29]. It has recently been shown that genetic selection of *P. pinaster* genotypes less susceptible to the pathogen could be an adequate measure to reduce the impact of the disease [30]. Moreover, the maternal abiotic environment in this pine species can determine seed mass, germination and early performance of the progeny [12], [13]. Following a logical rationale, we tested whether environmental maternal effects in response to abiotic factors may also influence seedling resistance to a plant pathogen.

## Materials and Methods

### Ethics Statement

Dirección Xeral de Montes (Xunta de Galicia) issued the permission for sampling. For fungal isolation and plant cultivation, no specific permissions were required for these activities. The field study (sampling) did not involve endangered or protected species.

### Plant and Fungal Material

Seed material was obtained from twin clonal *P. pinaster* seed orchards established within the Galician Maritime Pine Breeding Programme (Consellería do Medio Rural, Xunta de Galicia) to provide seeds of high genetic quality for reforestation in the area. In this programme, 116 unrelated genotypes selected for superior growth and stem form along the Atlantic coast of Galicia were included in each plantation (see [31] for details). The genotypes selected were clonally replicated by grafting and 10 copies of each genotype were planted at each site following a randomised complete block design with 10 blocks and single-tree plots. The two plantations included exactly the same genetic material, followed identical experimental designs, and were planted several km far from other *P. pinaster* stands to minimise pollen introgression. The seed orchards differ greatly, however, in site qualities. One is sited at Sergude (42.82°N, 8.45°W), in a favourable maternal environment for *P. pinaster* in terms of growth and reproduction rate, with mild temperatures, adequate moisture throughout the year and well drained, deep soil. The Monfero seed orchard (43.52°N, 7.93°W), however, is sited in an unfavourable maternal environment for *P. pinaster*, with low winter and spring temperatures, extreme wind exposure, and thin soil exposed to waterlogging (Supplementary Table S1).

For the present study, seeds were collected from 10 of the 116 genotypes included in each seed orchard. In January 2009, two cones from each of three individual trees per genotype and environment (ramets) were sampled. Cones were oven-dried at 35°C and all seeds were removed. Twelve randomly selected filled seeds per cone were used. Seeds were individually weighed (±0.0001 g) and stored at 4°C until sowing. A total of 1440 seeds (2 maternal environments ×10 genotypes ×3 ramets ×2 cones ×12 seeds) were sown.

The *F. circinatum* strain used (MAT-2) was isolated in May 2011 from a stem canker on a *P. radiata* tree in Cantabria, northern Spain (43.21°N, 4.43°W). Species identification was based on morphology and confirmed using molecular techniques after the virulence of the strain had been tested. As *F. circinatum* virulence in Spain is fairly homogeneous [32] and because different *F. circinatum* strains do not reveal significantly different rankings of susceptibility among the same host genotypes [33], [34], a single isolate was used.

### Greenhouse Experiment Design and Seedling Assessment

In April 2010, pre-weighed seeds were sown in 2 l pots containing a commercial substrate of sandy soil and peat (4:1 v/v, pH 5.8–6.8) and covered with a thin layer of sterilised white sand. Pots were set in a factorial design of 12 blocks, each including one plant from each of the 120 cones, with both the maternal environment and the maternal genotype randomly distributed within each block. Seedlings were grown in a greenhouse at Universidad de Extremadura (Plasencia, 40.03°N, 6.08°W), with temperatures fluctuating in the range of 23±5°C, under 70% full sunlight, and were watered every 2–3 days as necessary. Individual seedling height was measured monthly and individual stem diameter at ground level was measured when plant material was 18 months old.

### Fungal Inoculation and Symptom Assessments

On 17 October 2011, when plant material was 18 months old and 47.0±0.3 cm tall, half the blocks were inoculated with *F. circinatum* and the other half were mock inoculated with distilled sterile water to serve as control. On the day of inoculation, a spore suspension was prepared by firstly flooding *F. circinatum* cultures growing on PDA plates with a sterilised aqueous solution of 0.5 g KCl l^–1^ and then gently scraping the fungus from the surface with a sterile glass slide. The suspended fungal biomass was filtered through a double layer of sterile cheesecloth that retained mycelium and allowed most of the spores to pass through. The concentration of spores in the suspension was estimated using a hematocytometer and adjusted to a final concentration of 5·10^3^ spores ml^−1^ [35]. Inoculation was performed according to [36]. Small wounds, deep enough to reach the sapwood, were made at the junction of lignified and succulent tissue on the main stem using a drill bit (1.5 mm diameter) and 5 µl of the spore suspension (equivalent to approximately 25 spores) were placed in the wound site [34]. Control inoculations were performed by depositing distilled sterile water in the wound site instead of the spore suspension. Each tree was inoculated once.

Four weeks after inoculation, seedlings were examined. Bark was removed from the area surrounding the inoculation point and the length of the lesion on the sapwood was measured to the nearest mm (Figure S1). When necrosis girdled the stem, lesion length was estimated by measuring only necrosis proximal to the inoculation site, doubling this value as suggested by [37]. Seedlings with lesion length less than or equal to the length observed in control plants (∼0.3 cm) were considered not susceptible. Seedlings that had not been inoculated with the pathogen were studied following exactly the same procedures.

To confirm the presence of the fungus in the inoculated seedlings, *F. circinatum* from a random subsample of 25% of the seedlings harvested was re-isolated. A 5-cm segment near the point of inoculation was cut from each stem. Needles were removed and the stem surface was disinfested firstly in an aqueous solution of 0.1% (v/v) Tween® 20 (Panreac, Spain), then 70% (v/v) ethanol for 30 s and finally 20% (v/v) bleach for 1 minute. Each segment was plated onto FSM agar [38] and incubated at 22°C for 5–10 days. Colonies of *F. circinatum* were identified morphologically [39].

### Statistical Analysis

The effects of design factors on seedling growth (monthly seedling heights and stem diameters) were analysed with a general linear mixed model using the SAS PROC-MIXED procedure. For height growth analysis, we first attempted to fit a repeated measures mixed model, but this failed to converge. We then analysed each monthly height separately using a hierarchical model similar to those used to solve a split-split design, with three levels of nested experimental units (ramets, cones and seeds) [40]. Values within a single cone and values from different cones on the same ramet were assumed to be dependent measures within the same subject (cone or ramet, respectively) and were considered random factors. The general mixed model included the fixed effects of the maternal environment (E) and the block of the field experimental design nested within each seed orchard (B(E)). The random effects of the mixed model were i) maternal genotypes (G), ii) interaction between maternal genotypes and maternal environments (G×E, representing genetic variation in plastic responses to the maternal environment), and iii) the random effects of ramets (R) and cones within ramets (cone(R)), accounting for micro-environmental variation at scales lower than block size and other phenotypic effects associated with individual ramets and cones. The mixed model also included the fixed effect of the greenhouse blocks (trays) and the covariation with germination time, to account for greenhouse heterogeneity and variation in ontogenic development among seedlings, respectively.

For necrosis length analysis we used a general mixed model similar to the one just described, adding the fixed effect of the inoculation treatment (I) and its interaction with all the fixed and random factors indicated above. The I×E interaction indicates whether the maternal environment influences necrosis length after inoculation; i.e., whether there are significant maternal effects on seedling resistance to the pathogen. The I×G interaction random term accounts for genetic variation in susceptibility to the pathogen, whereas the I×G×E interaction was interpreted as genetic variation in the transmission of environmental maternal effects. Germination time was included again as a fixed covariate, but was removed as it was not significant and did not improve the resolution of the model.

As seed size differed between the maternal environments due to contrasting site quality (see details in Table S2 and Figure S2), all the previous models were run both including and excluding the individual seed mass as a covariate in the model to properly quantify the extent to which the maternal environmental effects observed were mediated by seed provisioning.

The statistical significance of the variance components for each random factor in all statistical models was assessed using likelihood ratio tests, where the differences in twice the log-likelihood of the models including or excluding each random factor were distributed as one tailed χ^2^, with one degree of freedom [41].

## Results

### Environmental Maternal Effects on Seedling Performance

Seed weight was strongly influenced by the contrasting site qualities of the maternal environments and the maternal genotypes (Table S2). Seeds from the favourable environment were 34% heavier than seeds from the unfavourable environment and differences were similar for the 10 genotypes studied (Figure S2).

**Figure 1:**
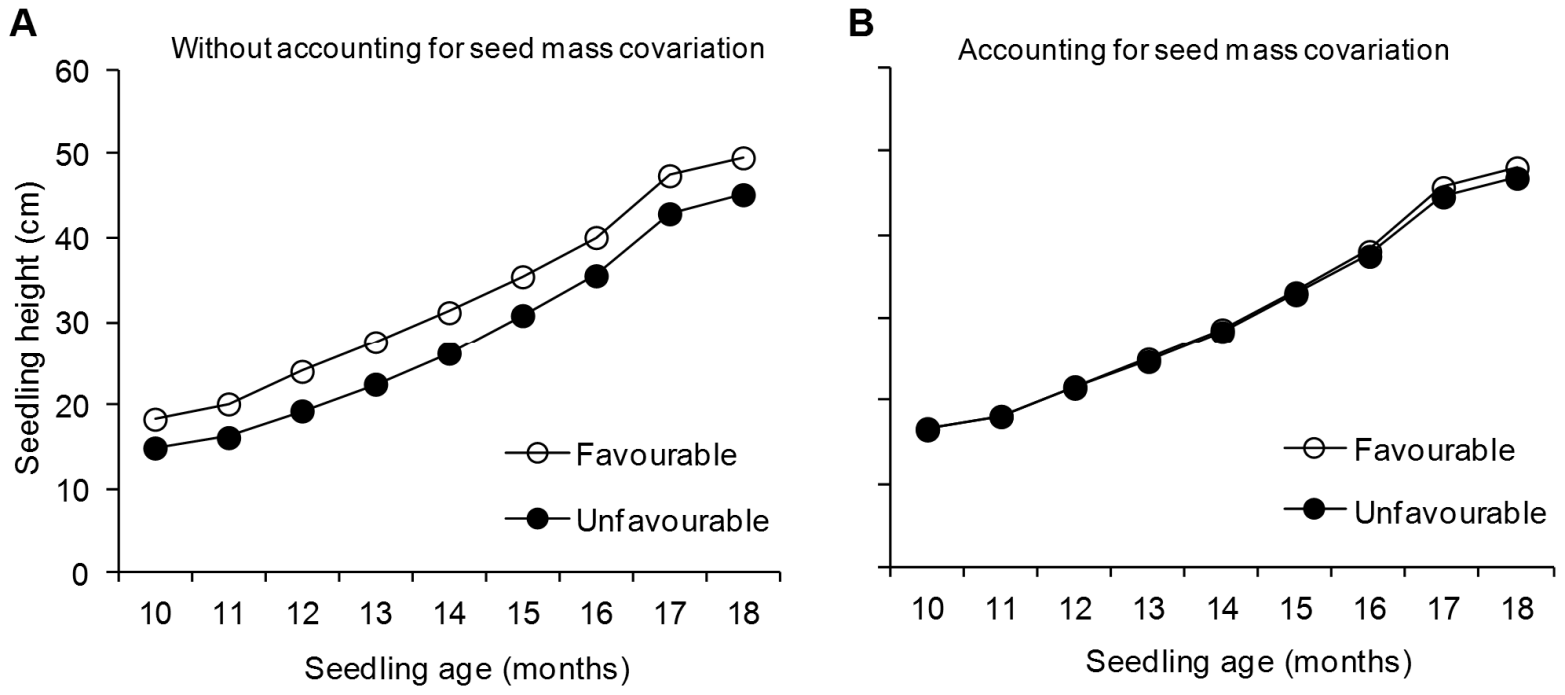
Changes in total height of *Pinus pinaster* seedlings. Seedlings were derived from 10 maternal genotypes clonally replicated in two contrasting maternal environments, one favourable (open circles) and one unfavourable (black circles) for pine growth and reproduction (N = 720). Least square means obtained from the mixed models excluding (A) and including (B) seed mass as a covariate are shown.

Without adjusting for seed mass covariation, seedling height was significantly influenced by both maternal environment and maternal genotype (Table 1-left). The effect of the maternal environment (Figure 1A) and the maternal genotype on seedling height was significant throughout the study period, although the magnitude of the effects diminished with seedling age, as seen in the decreasing *F* ratios (Table S3). At age 18 months, the average height of seedlings from the favourable maternal environment was 10.6% greater than those from the unfavourable maternal environment (Figure 1A) and seedling height ranged from 43.4 to 49.4 cm between maternal genotypes (Figure S3). Stem diameter was also larger in seedlings from the favourable maternal environment (*F*_1,9_ = 15.3; *P*<0.01), but no differences between genotypes were observed (χ^2^ = 1.4; *P*>0.05).

**Table 1:** Results of the general linear mixed model for analysis of the *Pinus pinaster* seedlings height 18 months after sowing.

Effects	Without accounting for seed mass covariation			Accounting for seed mass covariation		
	DF/VarComp	*F*-ratio/χ^2^	*P* value	DF/VarComp	*F*-ratio/χ^2^	*P* value
*Fixed factors*						
Maternal environment [E]	1, 9	20.2	**0.001**	1, 9	0.8	0.387
Block (E)^a^	10, 30	0.7	0.731	10, 30	0.7	0.747
Tray	11, 1206	27.7	**<0.001**	11, 1205	28.3	**<0.001**
Germination time	1, 1206	19.2	**<0.001**	1, 1205	16.1	**<0.001**
Seed mass				1, 1205	30.8	**<0.001**
*Random factors*						
Maternal genotype [G]	1.4±1.4	4.1	**0.021**	0.9±1.5	3.9	**0.024**
G×E	0.5±1.4	0.1	0.376	1.8±1.6	2.5	0.057
Ramet [R]	2.1±1.5	2.9	**0.044**	0.6±1.1	0.3	0.292
Cone (R)^a^	1.2±1.1	1.4	0.118	1.2±1.1	1.7	0.096
Residual	53.7±2.2			52.8±2.2		

Seedlings were derived from 10 maternal genotypes clonally replicated in two contrasting maternal environments, one favourable and one unfavourable for pine growth and reproduction. Analyses excluding and including individual seed mass as a covariate are shown. Degrees of freedom (DF) and *F*-ratios of fixed factors, and variance components (VarComp) and associated χ^2^ of random factors are shown. Significance (*P* value) is indicated in bold (*P*<0.05).

a Block was nested within maternal environment and cone was nested within ramet.

The effect of maternal environment on seedling height and diameter became negligible after including the covariation with the seed mass of each individual seedling in the mixed model (Table 1-right; Figure 1B). This result was consistently observed throughout the study period (Table S3). When adjusting for seed mass covariation, genetic variance for height growth decreased, although it remained significant on all assessment dates (Table 1-right; Table S3). The interaction between maternal environments and maternal genotypes for height growth was not significant, irrespective of whether seed weight covariation was taken into account (Table 1).

### Environmental Maternal Effects on Early Susceptibility to Pitch Canker Disease

No seedlings died during the study and no control seedlings showed necrosis lengths greater than the 0.3 cm caused by the drill bit. Only 3% of inoculated seedlings had lesion lengths less than or equal to 0.3 cm and were considered non-susceptible (4% from the favourable and 2.4% from the unfavourable maternal environment).

**Figure 2:**
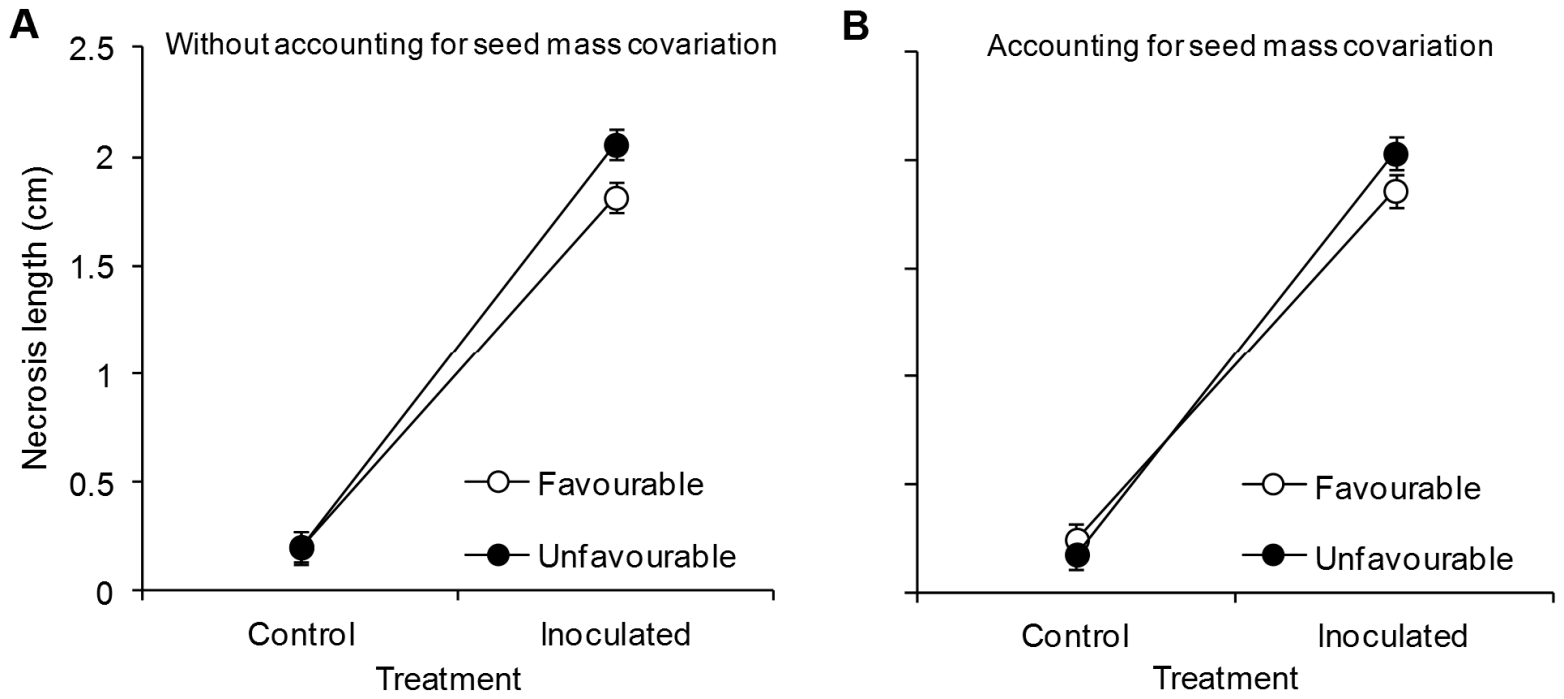
Necrosis length of *Pinus pinaster* seedlings caused by inoculation treatments. Seedlings were derived from 10 maternal genotypes clonally replicated in two contrasting maternal environments, one favourable (open circles) and one unfavourable (black circles) for pine growth and reproduction, four weeks after inoculation with the *Fusarium circinatum* pathogen (Inoculated) or mock inoculation with distilled sterile water (Control) (N = 360). Least square means ± standard errors obtained from the mixed models excluding (A) and including (B) seed mass as a covariate are shown.

**Figure 3:**
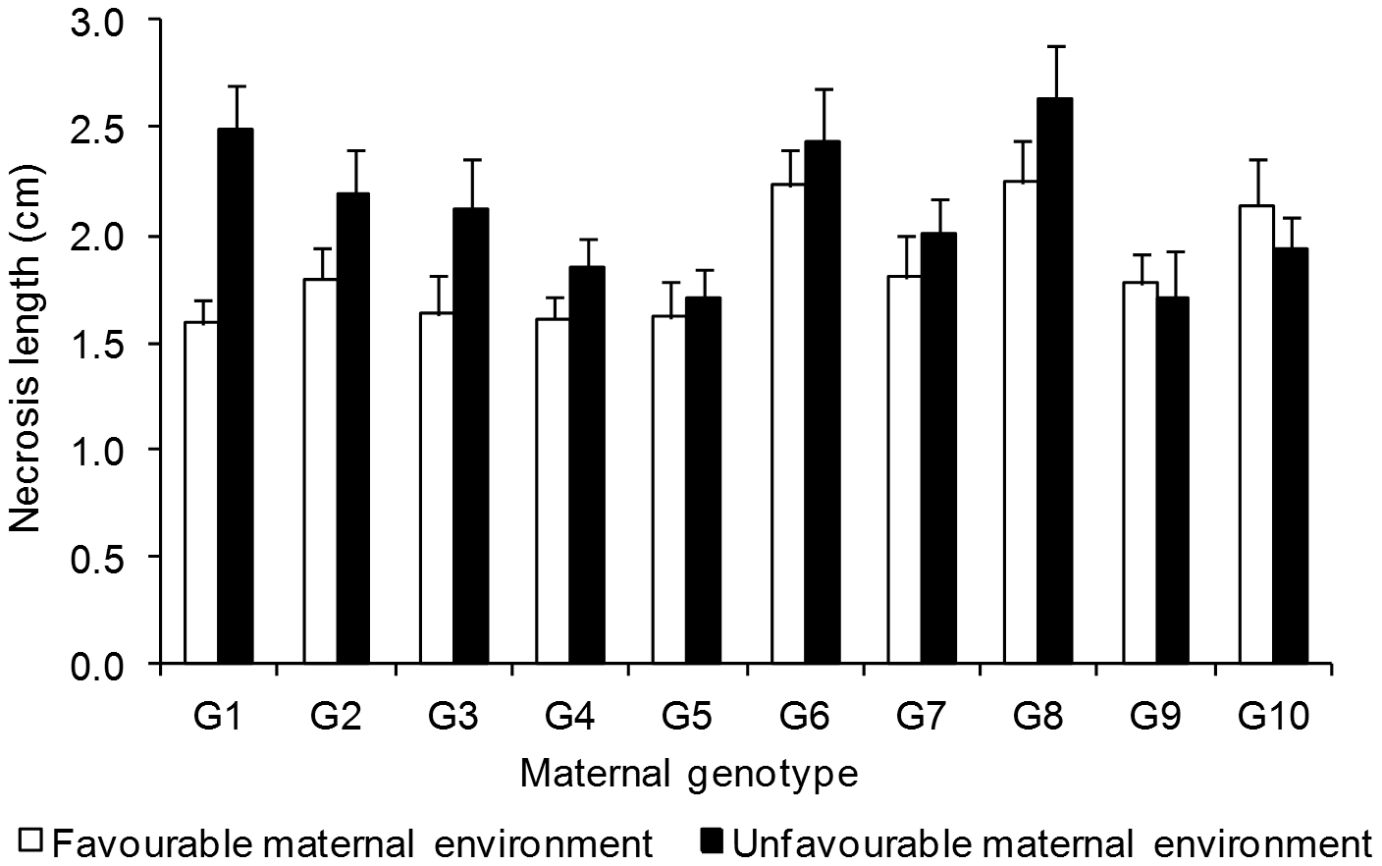
Mean necrosis length of *Pinus pinaster* seedlings caused by inoculation treatments. Seedlings were derived from 10 genotypes clonally replicated in two contrasting maternal environments, one favourable (white bars) and one unfavourable (black bars) for pine growth and reproduction, four weeks after inoculation with the *Fusarium circinatum* pathogen. Means ± standard errors are shown (N = 36).

Mean necrosis length was 16% shorter in inoculated seedlings from the favourable maternal environment than in inoculated seedlings from the unfavourable environment (Figure 2A), ranging from 1.7±0.2 to 2.4±0.2 cm between maternal genotypes (Figure 3). Without taking seed weight covariation into account, maternal environment and maternal genotype significantly affected necrosis length, as seen in Figure 2A and the significant I×E and I×G interactions (Table 2-left). The I×G×E interaction was not significant (Table 2-left), suggesting that the effect of maternal environment on damage intensity was similar across the 10 maternal genotypes. Contrary to what occurred with the maternal imprint on growth traits, the effect of maternal environment on resistance to pitch canker disease was not removed when individual seed mass was taken as a covariate in the mixed model (Table 2-right). Necrosis length remained larger in seedlings obtained from seeds from the unfavourable maternal environment (Figure 2B). Variation in necrosis lengths among maternal genotypes was also significant after adjusting for seed mass covariation (I×G interaction in Table 2-right), and no genetic variation in sensitivity to maternal environment was observed (non-significant I×G×E interaction; Table 2-right).

**Table 2:** Results of the general linear mixed model for analysis of the *Pinus pinaster* seedlings necrosis length.

Effects	Without accounting for seed mass covariation			Accounting for seed mass covariation		
	DF/VarComp	*F-ratio*/χ^2^	*P* value	DF/VarComp	*F-ratio*/χ^2^	*P* value
*Fixed factors*						
Inoculation [I]	1, 1175	493.5	**<0.001**	1, 1175	501.2	**<0.001**
Maternal environment [E]	1, 9	3.5	0.095	1, 9	0.4	0.561
I×E	1, 1175	7.0	**0.008**	1, 1175	7.0	**0.010**
Block (E)^a^	10, 30	0.8	0.627	10, 30	0.7	0.694
Trays (I)^b^	1, 1175	32.0	**<0.001**	10, 1174	32.1	**<0.001**
Seed mass				1, 1174	2.21	0.137
*Random factors*						
Maternal genotype [G]	0.002±0.012	0.0	0.500	0	0.5	0.500
G×E	0.001±0.008	0.9	0.171	0.001±0.007	1.1	0.240
Ramet [R]	0	0.0	0.500	0	0.0	0.500
I×G	0.02±0.015	17.1	**<0.001**	0.019±0.011	17.1	**<0.001**
I×G×E	0.007±0.01	0.7	0.201	0.008±0.011	1.1	0.147
Cone (R)^a^	0	0.0	0.500	0	0.0	0.500
Residual	0.453±0.018			0.452±0.018		

Seedlings were derived from 10 maternal genotypes clonally replicated in two contrasting maternal environments, one favourable and one unfavourable for pine growth and reproduction, four weeks after inoculation with the *Fusarium circinatum* pathogen or mock inoculation with distilled sterile water. Analyses excluding and including individual seed mass as a covariate are shown. Interaction terms I×E and I×G account for maternal effects and genetic variation, respectively, in seedling resistance to the pathogen, whereas the I×G×E interaction accounts for genetic variation in transgenerational responses. Degrees of freedom (DF) and *F*-ratios of fixed factors, and variance components (VarComp) and associated χ^2^ of random factors are shown. Significance (*P* value) is indicated in bold (*P*<0.05).

a Block was nested within maternal environment and cone was nested within ramet.

b Tray was nested within inoculation treatment.

## Discussion

### Seedling Performance Variation

Abiotic conditions of the maternal environment influenced height and stem diameter of *P. pinaster* progenies for at least 1.5 years after sowing. Seedlings grown from favourable maternal environment seeds were taller and had thicker stems than seedlings grown from unfavourable maternal environment seeds and this trend was consistent among all genotypes. Earlier studies also identified larger offspring phenotypes from seeds from favourable maternal environments [18], [42]. Most studies (e.g. [9], [43]) showed that these maternal environmental effects on seedling performance were seed mass dependent and disappeared when the statistical model properly accounted for seed weight variation. Although maternal environmental effects explaining variations in seedling performance have previously been identified in conifers (e.g. [10], [44]), less attention has been paid to the interactive effects of maternal environment and maternal genotype (i.e., whether transgenerational responses are genetically variable) and to how long the mediation of seed provisioning in the transmission of maternal effects lasts. The influence of seed mass on *P. pinaster* seedling height became less pronounced with time, confirming that maternal environment effects mediated by seed provisioning diminish with age [20].

Seed mass is a key trait for many aspects of the ecology and evolution of plant species [45]. The quantity and quality of resources allocated to seeds may differ strongly between environments and this may result in large differences in mean seed weight among lots from different environments [8], as observed here. Some studies have shown positive relations between seed size and the probability and speed of germination [46], and between seed size and subsequent seedling size [47], [48]. Our results, showing a large difference in seedling growth depending on the environment where the seeds develop, could be explained by the influence of the resources allocated to seeds by mother trees. This influence was not genetically based (no G×E interaction).

### Pitch Canker Susceptibility Variation

The results showed that the plant damage caused by *F. circinatum* was largely influenced by maternal environment and maternal genotype. Interestingly, the influence of these factors was significant even after considering the differential seed provisioning in the model. This result suggests that transgenerational defensive plasticity to pitch canker disease in *P. pinaster* offspring was not a passive response of resource availability in seed provisioning. In the following, transgenerational responses will be referred to as the effects transmitted to a single generation.

Most studies examining transgenerational plastic responses of plants have focused on determining whether seedlings derived from mother plants growing under a particular biotic or abiotic stress are better able to tolerate that particular stress [16], [49], [50]. Transgenerational induction of defence to organisms in response to a different biotic stress has also been reported in several plant species [15]. However, transgenerational elicitation of genotypes with different resistance to an organism due to maternal exposure to a previous abiotic stressor is reported here for the first time. This finding suggests that the transgenerational resistance of pines due to an abiotic stress may interact (i.e. be cross-linked) with the defensive response of pines to a biotic stress.

Growth and resistance are not independent features of an individual plant, and usually there is a trade-off between the two [51]. Maximising growth and resistance at the same time is not possible, and plants tend to optimise resource allocation to each feature depending on environmental conditions [52]. Various theoretical frameworks predict that plants growing in resource-limited environments should be better protected against natural enemies (reviewed by [53]). Results reported here are not consistent with these predictions and indicate not only that bigger seedlings are more resistant, but also that the offspring of maternal trees growing under resource deprivation show reduced resistance. Moreover, earlier studies observed no negative genetic correlations between growth and resistance [30], [46], indicating that selection for growing *P. pinaster* trees in breeding programmes would not necessarily imply increased susceptibility to pathogens.

Seed mass had no statistical relation to seedling ability to resist the pathogen. Other non-exclusive transmission factors may be involved in this form of transgenerational plasticity. Seeds from the two contrasting maternal environments could, for example, differ qualitatively in their seed biochemistry. Differential allocation of defensive chemicals and/or defence-inducing hormones to seeds may influence seedling resistance [4]. Alternatively, epigenetic mechanisms, i.e. processes that modulate gene expression with no variations in DNA base sequences [21], could be involved. Epigenetic mechanisms are known to be responsible for different forms of transgenerational plasticity [4]. In particular, conifers have a large amount of genomic DNA without apparent duplications, which could be rich source sites for epigenetic regulation and modification [7]. Information about seed biochemistry composition and gene expression is not available, but the fact that the offspring response was independent of seed mass and seedling growth suggests that these traits could be involved in the plasticity observed over the next generation. As transgenerational changes in epigenetic regulation of gene expression can be long lasting [23], disentangling their role in the offspring responses would help to elucidate whether the differences observed in seedling resistance will be maintained as seedlings age.

Systemic induced resistance of *P. radiata* in response to *F. circinatum* infection has been demonstrated in greenhouse and field conditions [37]. However, it is not known whether induction of defences to pitch canker can be transmitted to the next generation or whether this would vary within genotypes. Earlier studies confirmed different levels of *P. pinaster* resistance to pitch canker disease [30]. We can now add that seedling resistance seems to be a plastic trait controlled by both environmental maternal effects and maternal genotype. On the one hand, environmental maternal effect increased the negative fitness consequences of the ‘poor’ environment. On the other hand, the lack of genetic variation in the transgenerational response in seedling resistance (i.e. non-significant G×E interaction) would indicate a possible constraint for evolution of this trait. Genetic variation in sensitivity to the maternal environment does not seem to exist within the population studied. This lack of variation contrasts with other experiments (e.g. [49]), probably because the seedlings used here, derived from selected improved trees, included only a small proportion of the total genetic variation of the species studied. The absence of co-evolution of the native pine species with the introduced microbe should also be taken into account.

### Conclusions

This study has demonstrated that abiotic differences in the maternal environment affected both plant growth and resistance to *F. circinatum* traits in the subsequent generation, up to 1.5 years after germination. Quantitative maternal investment in seed mass explained the transgenerational plasticity of seedling growth but not of seedling resistance to *F. circinatum*, in which other mechanisms must be involved. The *P. pinaster* progenies derived from mother trees growing in a favourable maternal environment in terms of growth and reproduction showed higher growth rates and improved resistance to the *F. circinatum* pathogen than progenies derived from mother trees growing in the unfavourable environment. From a practical point of view, we recommend examining the possibilities of transgenerational responses as drivers of seedling resistance and phenotypic performance in nurseries, breeding programmes, forests plantations and management practices.

## Supporting Information

Figure S1 **Necrosis length of** ***Pinus pinaster*** **seedlings four weeks after inoculation with the** ***Fusarium circinatum*** **pathogen.** Scale bar = 0.3 cm.
(TIF)

Figure S2 **Mean seed mass of** ***Pinus pinaster*** **seedlings.** Seedlings were derived from 10 maternal genotypes clonally replicated in two contrasting maternal environments, one favourable (white bars) and one unfavourable (black bars) for pine growth and reproduction. Means ± standard errors are shown (N = 72).
(TIF)

Figure S3 **Mean seedling height of** ***Pinus pinaster*** **seedlings.** Seedlings were derived from 10 maternal genotypes clonally replicated in two contrasting maternal environments, one favourable (white bars) and one unfavourable (black bars) for pine growth and reproduction. Means ± standard errors are shown (N = 72).
(TIF)

Table S1 **Climatic, edaphic and dasometric characteristics of the two contrasting maternal environments.**
(DOC)

Table S2 **Results of the general linear mixed model for analysis of individual seed mass of** ***Pinus pinaster*** **.**
(DOC)

Table S3 **Results of the general linear mixed model for analysis of monthly heights (February–October 2011) of the** ***Pinus pinaster*** **seedlings.**
(DOC)

## References

[1] Roach DA, Wulff RD (1987) Maternal effects in plants. Annu Rev Ecol Syst 18: 209–235.

[2] Donohue K (2009) Completing the cycle: maternal effects as the missing link in plant life histories. Philos Trans R Soc Lond B Biol Sci 364: 1059–1074. 19324611 10.1098/rstb.2008.0291 PMC2666684

[3] Galloway LF (2005) Maternal effects provide phenotypic adaptation to local environmental conditions. New Phytol 166: 93–100. 15760354 10.1111/j.1469-8137.2004.01314.x

[4] Herman JJ, Sultan SE (2011) Adaptive transgenerational plasticity in plants: case studies, mechanisms, and implications for natural populations. Front Plant Sci 2: 1–10. 22639624 10.3389/fpls.2011.00102 PMC3355592

[5] Galloway LF, Etterson JR (2007) Transgenerational plasticity is adaptive in the wild. Science 318: 1134–1136. 18006745 10.1126/science.1148766

[6] Whittle CA, Otto SP, Johnston MO, Krochko JE (2009) Adaptive epigenetic memory of ancestral temperature regime in *Arabidopsis thaliana* . Botany 87: 650–657.

[7] Yakovlev I, Fossdal CG, Skrøppa T, Olsen JE, Jahren AH, et al (2012) An adaptive epigenetic memory in conifers with important implications for seed production. Seed Sci Res 22: 63–76.

[8] Violle CH, Castro H, Richarte J, Navas ML (2009) Intraspecific seed trait variations and competition: passive or adaptive response? Funct Ecol 23: 612–620.

[9] Elwell AL, Gronwall DS, Miller ND, Spalding EP, Brooks TLD (2011) Separating parental environment from seed size effects on next generation growth and development in Arabidopsis. Plant Cell Environ 34: 291–301. 20955226 10.1111/j.1365-3040.2010.02243.x

[10] Stoehr MU, L'Hirondelle SJ, Binder WD, Webber JE (1998) Parental environment aftereffects on germination, growth, and adaptive traits in selected white spruce families. Can J For Res 28: 418–426.

[11] Webber J, Ott P, Owens J, Binder W (2005) Elevated temperature during reproductive development affects cone traits and progeny performance in *Picea glauca×engelmannii* complex. Tree Physiol 25: 1219–1227. 16076771 10.1093/treephys/25.10.1219

[12] Cendán C, Sampedro L, Zas R (2013) The maternal environment determines the timing of germination in *Pinus pinaster*. Environ Exp Bot. doi: 10.1016/j.envexpbot.2011.11.022.

[13] Zas R, Cendán C, Sampedro L (2013) Mediation of seed provisioning in the transmission of environmental maternal effects in Maritime pine (*Pinus pinaster* Aiton). Heredity doi: 10.1038/hdy.2013.44. 10.1038/hdy.2013.44 PMC3746824 23652562

[14] Skrøppa T, Tollefsrud M, Sperisen C, Johnsen Ø (2010) Rapid change in adaptive performance from one generation to the next in *Picea abies*–Central European trees in a Nordic environment. Tree Genet Genomes 6: 93–99.

[15] Holeski LM, Jander G, Agrawal AA (2012) Transgenerational defense induction and epigenetic inheritance in plants. Trends Ecol Evol 27: 618–626. 22940222 10.1016/j.tree.2012.07.011

[16] Agrawal AA (2002) Herbivory and maternal effects: Mechanisms and consequences of transgenerational induced plant resistance. Ecology 83: 3408–3415.

[17] Rasmann S, De Vos M, Casteel CL, Tian D, Halitschke R, et al (2012) Herbivory in the previous generation primes plants for enhanced insect resistance. Plant Physiol 158: 854–863. 22209873 10.1104/pp.111.187831 PMC3271773

[18] Castro J, Hódar JA, Gómez JM (2006) Seed size. In: Basra AS, editor. Handbook of seed science and technology (seed biology, production, and technology). New York: Harworth Press. 397–428.

[19] Metz J, Liancourt P, Kigel J, Harel D, Sternberg M, et al (2010) Plant survival in relation to seed size along environmental gradients: a long-term study from semi-arid and Mediterranean annual plant communities. J Ecol 98: 697–704.

[20] Boyko A, Kovalchuk I (2011) Genome instability and epigenetic modification - heritable responses to environmental stress? Curr Opin Plant Biol 14: 260–266. 21440490 10.1016/j.pbi.2011.03.003

[21] Jablonka E, Raz G (2009) Transgenerational epigenetic inheritance: prevalence, mechanisms, and implications for the study of heredity and evolution. Q Rev Biol 84: 131–176. 19606595 10.1086/598822

[22] Holeski LM (2007) Within and between generation phenotypic plasticity in trichome density of *Mimulus guttatus* . J Evol Biol 20: 2092–2100. 17903186 10.1111/j.1420-9101.2007.01434.x

[23] Herman JJ, Sultan SE, Horgan-Kobelski T, Riggs C (2012) Adaptive transgenerational plasticity in an annual plant: grandparental and parental drought stress enhance performance of seedlings in dry soil. Integr Comp Biol 52: 77–88. 22523124 10.1093/icb/ics041

[24] Rapp RA, Wendel JF (2005) Epigenetics and plant evolution. New Phytol 168: 81–91. 16159323 10.1111/j.1469-8137.2005.01491.x

[25] Santini A, Ghelardini L, De Pace C, Desprez-Loustau ML, Capretti P, et al (2013) Biogeographical patterns and determinants of invasion by forest pathogens in Europe. New Phytol 197: 238–250. 23057437 10.1111/j.1469-8137.2012.04364.x

[26] Wingfield MJ, Hammerbacher A, Ganley RJ, Steenkamp ET, Gordon TR, et al (2008) Pitch canker caused by *Fusarium circinatum* - a growing threat to pine plantations and forests worldwide. Australas Plant Pathol 37: 319–334.

[27] Landeras E, García P, Fernández Y, Braña M, Fernández-Alonso O, et al (2005) Outbreak of pitch canker caused by *Fusarium circinatum* on *Pinus* spp. in northern Spain. Plant Dis 89: 1015. 10.1094/PD-89-1015A 30786652

[28] Berra D, Urkola A (2010) Epidemiología de *Fusarium circinatum* en plantaciones forestales de Gipuzkoa. In: Sociedad Española de Fitopatología, editors. Proceedings of the XV Congreso de la Sociedad Española de Fitopatología. Vitoria-Gasteiz: Spain. 282.

[29] Vivas M, Martín JA, Gil L, Solla A (2012) Evaluating methyl jasmonate for induction of resistance to *Fusarium oxysporum*, *F. circinatum* and *Ophiostoma novo-ulmi* . For Syst 21: 289–299.

[30] Vivas M, Zas R, Solla A (2012) Screening of Maritime pine (*Pinus pinaster*) for resistance to *Fusarium circinatum*, the causal agent of Pitch Canker disease. Forestry 25: 185–192.

[31] Zas R, Merlo E, Fernández-López J (2004) Genotype x environment interaction in Maritime pine families in Galicia, Northwest Spain. Silvae Genet 53: 175–182.

[32] Iturritxa E, Ganley RJ, Wright J, Heppe E, Steenkamp ET, et al (2011) A genetically homogenous population of *Fusarium circinatum* causes pitch canker of *Pinus radiata* in the Basque Country, Spain. Fungal Biol 115: 288–295. 21354535 10.1016/j.funbio.2010.12.014

[33] Gordon TR, Wikler KR, Clark SL, Okamoto D, Storer AJ, et al (1998) Resistance to pitch canker disease, caused by *Fusarium subglutinans* f. sp. *pini* in Monterey pine (*Pinus radiata*). Plant pathol 47: 706–711.

[34] Matheson AC, Devery ME, Gordon TR, Werner W, Vogler DR, et al (2006) Heritability of response to inoculation by pine pitch canker of seedlings of radiata pine. Australas For 69: 101–106.

[35] Schmale DG, Gordon TR (2003) Variation in susceptibility to pitch canker disease, caused by *Fusarium circinatum*, in native stands of *Pinus murcicata* . Plant Pathol 52: 720–725.

[36] Gordon TR, Okamoto D, Storer AJ, Wood DL (1998) Susceptibility of five landscape pines to pitch canker disease, caused by *Fusarium subglutinans* f. sp. *pini* . Hortscience 33: 868–871.

[37] Gordon TR, Kirkpatrick SC, Aegerter BJ, Fisher AJ, Storer AJ, et al (2011) Evidence for the occurrence of induced resistance to pitch canker, caused by *Gibberella circinata* (anamorph *Fusarium circinatum*), in populations of *Pinus radiata* . Forest Pathol 41: 227–232.

[38] Aegerter BJ, Gordon TR (2006) Rates of pitch canker induced seedling mortality among *Pinus radiata* families varying in levels of genetic resistance to *Gibberella circinata* (anamorph *Fusarium circinatum*). For Ecol Manage 235: 14–17.

[39] Leslie JF, Summerell BA (2006) The *Fusarium* laboratory manual. Iowa: Blackwell Publishing. 388 p.

[40] Littell RC, Milliken GA, Stroup WW, Wolfinger RD, Schabenberger O (2006) SAS System for mixed models. North Carolina: SAS Institute. 647 p.

[41] Fry JD (2004) Estimation of genetic variances and covariances by restricted maximum likelihood using PROC MIXED. In: Saxton AM, editor. Genetic analysis of complex traits using SAS. North Carolina: SAS Institute. 11–34.

[42] Leishman MR, Wright IJ, Moles AT, Westoby M (2000) Seeds: the ecology of regeneration in plant communities. In: Fenner M, editor. The evolutionary ecology of seed size: CABI, Wallingford. 31–57.

[43] Castro J (1999) Seed mass versus seedling performance in Scots pine: a maternally dependent trait. New Phytol 144: 153–161.

[44] Lindgren D, Wei R-P (1994) Effects of maternal environment on mortality and growth in young *Pinus sylvestris* in field trials. Tree Physiol 14: 323–327. 14967706 10.1093/treephys/14.3.323

[45] Linkies A, Graeber K, Knight C, Leubner-Metzger G (2010) The evolution of seeds. New Phytol 186: 817–831. 20406407 10.1111/j.1469-8137.2010.03249.x

[46] Solla A, Aguín O, Cubera E, Sampedro L, Mansilla JP, et al (2011) Survival time analysis of *Pinus pinaster* inoculated with *Armillaria ostoyae*: genetic variation and relevance of seed and root traits. Eur J Plant Pathol 130: 477–488.

[47] Weiner J, Martínez S, Mueller-Schaerer H, Stoll P, Schmid B (1997) How important are environmental maternal effects in plants? A study with *Centaurea maculosa* . J Ecol 85: 133–142.

[48] Moles AT, Westoby M (2006) Seed size and plant strategy across the whole life cycle. Oikos 113: 91–105.

[49] Agrawal AA (2001) Transgenerational consequences of plant response to herbivory: an adaptive maternal effect. Am Nat 157: 555–569. 18707262 10.1086/319932

[50] Latzel V, Klimešová J, Hájek T, Gómez S, Šmilauer P (2010) Maternal effects alter progeny's response to disturbance and nutrients in two *Plantago* species. Oikos 119: 1700–1710.

[51] Koricheva J (2002) Meta-analysis of sources of variation in fitness costs of plant antiherbivore defenses. Ecology 83: 176–190.

[52] Herms DA, Mattson WJ (1992) The dilemma of plants: to grow or defend. Q Rev Biol 67: 283–335.

[53] Stamp N (2003) Out of the quagmire of plant defense hypotheses. Q Rev Biol 78: 23–55. 12661508 10.1086/367580

