# Supplementary figures and images for: Environmental Maternal Effects Mediate the Resistance of Maritime Pine to Biotic Stress
Source: PLoS One. 2013 Jul 26;8(7):e70148. doi: 10.1371/journal.pone.0070148 (PMC3724826; doi:10.1371/journal.pone.0070148)

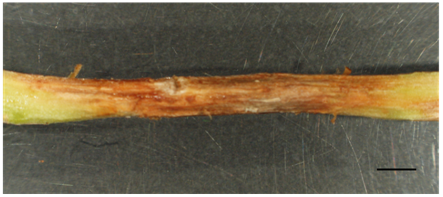

Supplement: Figure S1 — Necrosis length of Pinus pinaster seedlings four weeks after inoculation with the Fusarium circinatum pathogen. Scale bar = 0.3 cm. (TIF) [file pone.0070148.s001.tif]

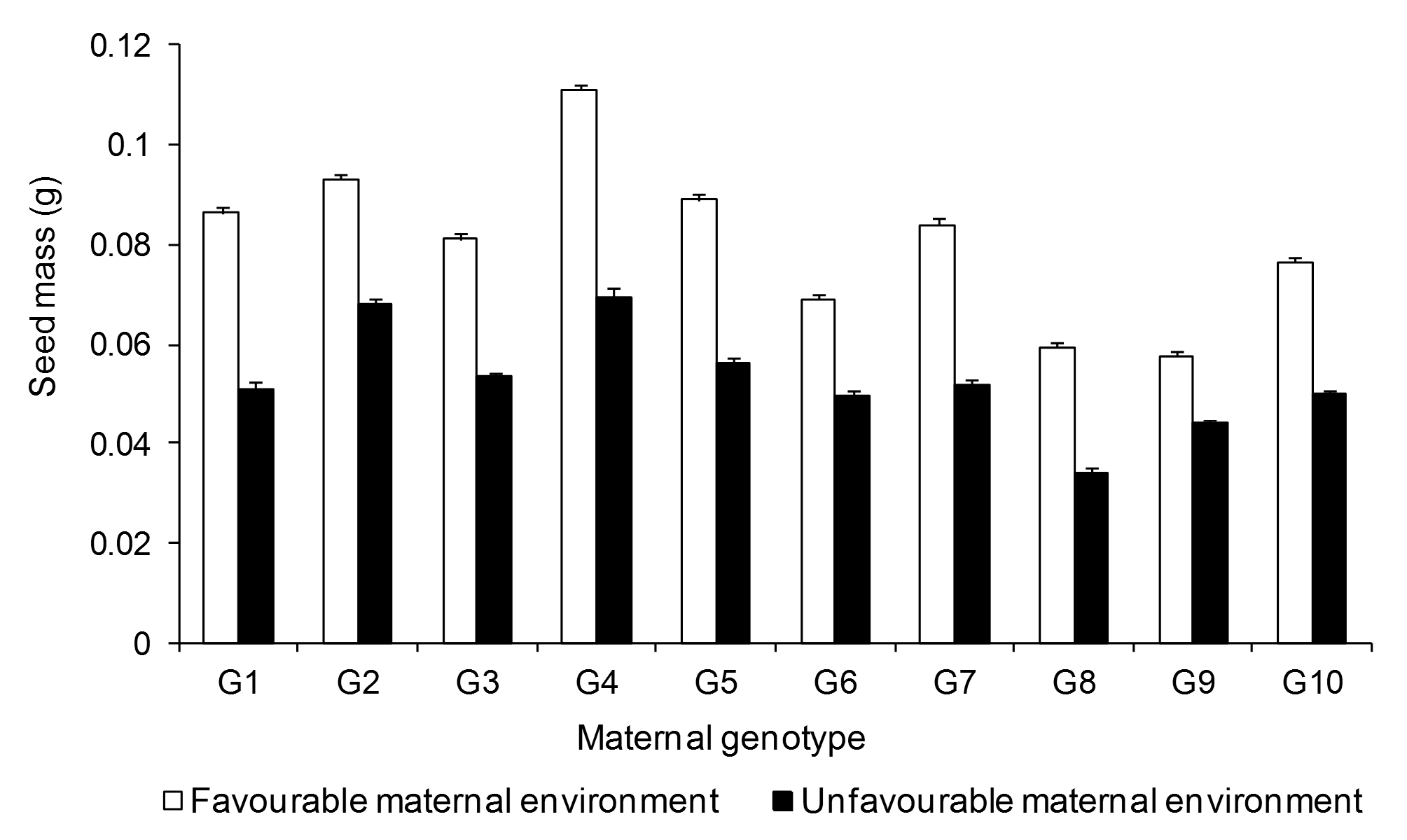

Supplement: Figure S2 — Mean seed mass of Pinus pinaster seedlings. Seedlings were derived from 10 maternal genotypes clonally replicated in two contrasting maternal environments, one favourable (white bars) and one unfavourable (black bars) for pine growth and reproduction. Means ± standard errors are shown (N = 72). (TIF) [file pone.0070148.s002.tif]

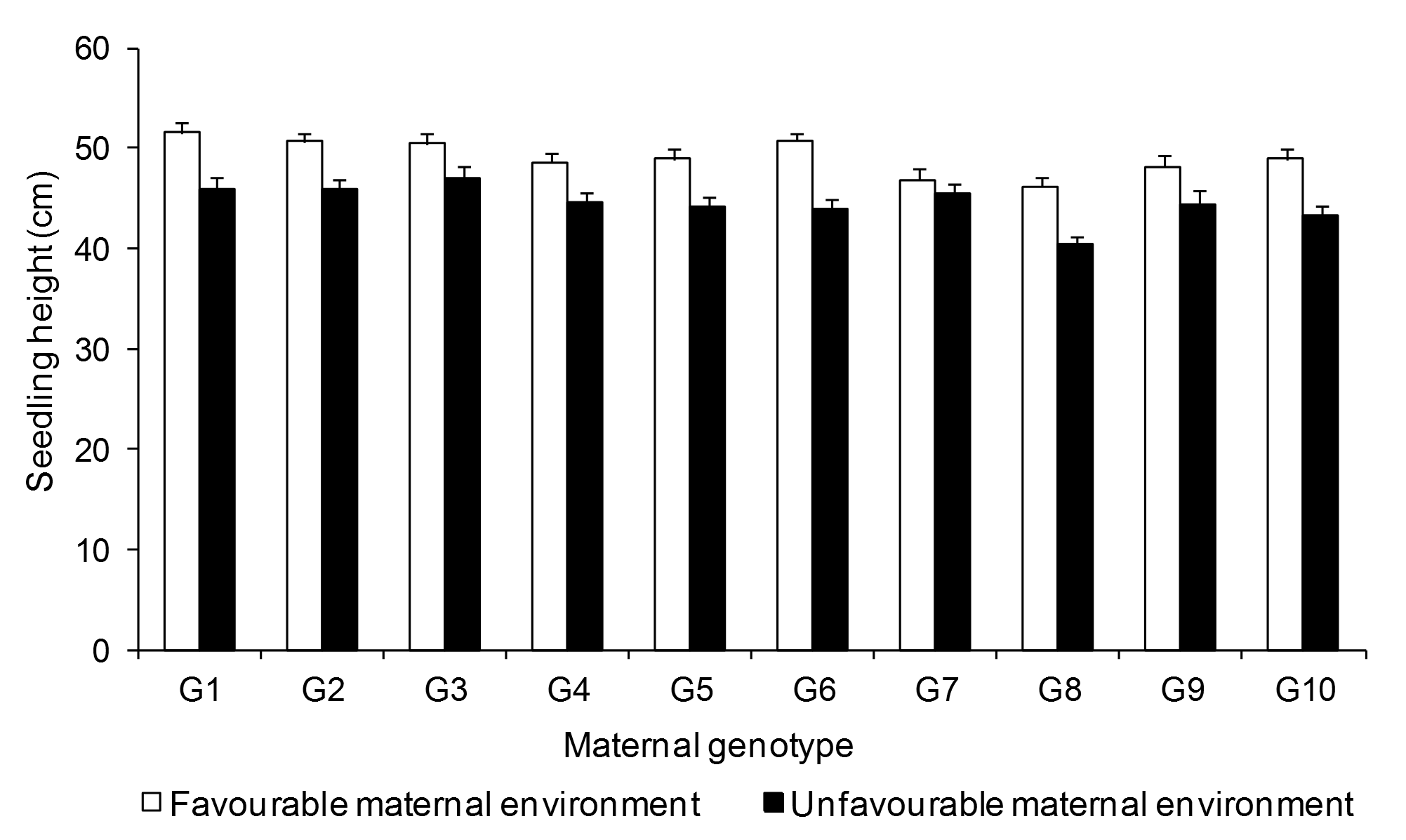

Supplement: Figure S3 — Mean seedling height of Pinus pinaster seedlings. Seedlings were derived from 10 maternal genotypes clonally replicated in two contrasting maternal environments, one favourable (white bars) and one unfavourable (black bars) for pine growth and reproduction. Means ± standard errors are shown (N = 72). (TIF) [file pone.0070148.s003.tif]
