# Supplementary material for: Environmental Maternal Effects Mediate the Resistance of Maritime Pine to Biotic Stress
Source: PLoS One. 2013 Jul 26;8(7):e70148. doi: 10.1371/journal.pone.0070148 (PMC3724826; doi:10.1371/journal.pone.0070148)
Supplement: Table S1 — Climatic, edaphic and dasometric characteristics of the two contrasting maternal environments. (DOC) [file pone.0070148.s004.doc]

**Table S1.** Climatic, edaphic and dasometric characteristics of the two contrasting maternal environments.

|  | Sergude | Monfero |
| --- | --- | --- |
| Altitude (m) | 258 | 615 |
| Mean annual temperature (ºC) | 13.2 | 10.6 |
| Maximum temperature (ºC) | 38.7 | 35.3 |
| Minimum temperature (ºC) | -4.7 | -8.0 |
| Mean temperature of the warmest month (ºC) | 19.2 | 15.5 |
| Mean temperature of the coldest month (ºC) | 7.8 | 5.9 |
| Number of frost-free months ^a^ | 5 | 3 |
| Annual precipitation (l m^-2^) | 1445 | 1435 |
| Daily average wind speed (m s^-1^) | 3.2 | 5.2 |
| Number of windy days per year ^b^ | 35 | 166 |
| Soil pH (H_2_O, 1:2.5) | 5.1 | 4.5 |
| Soil depth (cm) | >120 cm | 45.1 ± 3.2 |
| Tree age at sampling | 27 | 20 |
| Mean tree diameter at breast height (cm) | 20.9 ± 0.6 | 6.1 ± 0.3 |
| Annual individual tree growth in basal area (cm^2^year^-1^) | 13.6 ± 0.8 | 1.65 ± 0.15 |
| Number of cones per tree at age 9 | 76.5 ± 11.0 | 3.8 ± 0.2 |
| Reproductive allocation (cones dm^-2^) ^c^ | 47.8 ± 4.1 | 15.8 ± 2.8 |

Characteristics of the two contrasting maternal environments, one favourable (Sergude) and one unfavourable (Monfero) for pine growth and reproduction. Both seed orchards have exactly the same genetic material and experimental spatial design (Cendán et al. 2013).

^a^ *Sensu* Emberger et al. (1963); i.e., the period when the average minimum temperature is more than 7ºC

^b^ Average wind speed > 5 m s^-1^

^c^ Number of cones per unit of basal area at breast height (modified from Climent et al. 2008).
