# Supplementary material for: Environmental Maternal Effects Mediate the Resistance of Maritime Pine to Biotic Stress
Source: PLoS One. 2013 Jul 26;8(7):e70148. doi: 10.1371/journal.pone.0070148 (PMC3724826; doi:10.1371/journal.pone.0070148)
Supplement: Table S2 — Results of the general linear mixed model for analysis of individual seed mass of Pinus pinaster . (DOC) [file pone.0070148.s005.doc]

**Table S2.** Results of the general linear mixed model for analysis of individual seed mass of *Pinus pinaster*.

| Effects | | Seed mass | | |
| --- | --- | --- | --- | --- |
|  |  | DF / VarComp | *F*-ratio / χ^2^ | *P* value |
| *Fixed factors* | |  |  |  |
|  | Maternal environment [E] | 1, 9 | 67.7 | < 0.001 |
|  | Block(E) ^a^ | 10, 30 | 4.2 | 0.001 |
|  |  |  |  |  |
| *Random factors* | |  |  |  |
|  | Maternal genotype [G] | 138.6 ± 76.6 | 42.7 | < 0.001 |
|  | G × E | 30.9 ± 2.1 | 6.1 | 0.007 |
|  | Ramet [R] | 15.9 ± 8.9 | 4.8 | 0.014 |
|  | Cone (R) ^a^ | 29.9 ± 6.1 | 319.8 | < 0.001 |
|  | Residual | 39.6 ± 1.5 |  |  |

Seedlings were derived from 10 maternal genotypes clonally replicated in two contrasting maternal environments, one favourable and one unfavourable for pine growth and reproduction. Degrees of freedom (DF) and *F*-ratios of fixed factors, and variance components (VarComp) and associated χ^2^ of random factors are shown.

^a^ Block was nested within maternal environment and cone was nested within ramet.
