# Supplementary material for: Environmental Maternal Effects Mediate the Resistance of Maritime Pine to Biotic Stress
Source: PLoS One. 2013 Jul 26;8(7):e70148. doi: 10.1371/journal.pone.0070148 (PMC3724826; doi:10.1371/journal.pone.0070148)
Supplement: Table S3 — Results of the general linear mixed model for analysis of monthly heights (February–October 2011) of the Pinus pinaster seedlings. (DOC) [file pone.0070148.s006.doc]

**Table S3.** Results of the general linear mixed model for analysis of monthly heights (February - October 2011) of the *Pinus pinaster* seedlings.

1. Without accounting for seed mass covariation

| Effects | |  | February | |  | March | |  | April | |  | May | |  | June | |  | July | |  | August | |  | September | |  | October | |
| --- | --- | --- | --- | --- | --- | --- | --- | --- | --- | --- | --- | --- | --- | --- | --- | --- | --- | --- | --- | --- | --- | --- | --- | --- | --- | --- | --- | --- |
|  |  | DF | *F*/χ^2^ | *P* value |  | *F*/χ^2^ | *P* value |  | *F*/χ^2^ | *P* value |  | *F*/χ^2^ | *P* value |  | *F*/χ^2^ | *P* value |  | *F*/χ^2^ | *P* value |  | *F*/χ^2^ | *P* value |  | *F*/χ^2^ | *P* value |  | *F*/χ^2^ | *P* value |
| *Fixed factors* | |  |  |  |  |  |  |  |  |  |  |  |  |  |  |  |  |  |  |  |  |  |  |  |  |  |  |  |
|  | Maternal environment [E] | 1, 9 | 38.4 | **< 0.001** |  | 38.5 | **< 0.001** |  | 38.9 | **< 0.001** |  | 37.6 | **< 0.001** |  | 36.8 | **< 0.001** |  | 29.4 | **< 0.001** |  | 25.5 | **< 0.001** |  | 21.0 | **0.001** |  | 20.2 | **0.001** |
|  | Block(E) ^a^ | 10, 30 | 1.7 | 0.134 |  | 1.1 | 0.394 |  | 0.8 | 0.597 |  | 0.9 | 0.572 |  | 0.9 | 0.57 |  | 0.8 | 0.600 |  | 0.8 | 0.653 |  | 0.7 | 0.723 |  | 0.7 | 0.731 |
|  | Tray | 11, 1206 | 42.1 | **< 0.001** |  | 35.5 | **< 0.001** |  | 26.0 | **< 0.001** |  | 26.9 | **< 0.001** |  | 30.7 | **< 0.001** |  | 34.3 | **< 0.001** |  | 37.7 | **< 0.001** |  | 26.0 | **< 0.001** |  | 27.7 | **< 0.001** |
|  | Germination time | 1, 1206 | 58.1 | **< 0.001** |  | 59.7 | **< 0.001** |  | 62.3 | **< 0.001** |  | 67.4 | **< 0.001** |  | 57.0 | **< 0.001** |  | 41.1 | **< 0.001** |  | 27.0 | **< 0.001** |  | 23.4 | **< 0 .001** |  | 19.2 | **< 0.001** |
|  |  |  |  |  |  |  |  |  |  |  |  |  |  |  |  |  |  |  |  |  |  |  |  |  |  |  |  |  |
| *Random factors* | |  |  |  |  |  |  |  |  |  |  |  |  |  |  |  |  |  |  |  |  |  |  |  |  |  |  |  |
|  | Maternal genotype [G] |  | 27.5 | **< 0.001** |  | 24.2 | **< 0.001** |  | 21.0 | **< 0.001** |  | 19.8 | **< 0.001** |  | 18.7 | **< 0.001** |  | 13.3 | **< 0.001** |  | 8.0 | **0.002** |  | 4.4 | **0.018** |  | 4.1 | **0.021** |
|  | G × E |  | 0.0 | 0.500 |  | 0.0 | 0.500 |  | 0.0 | 0.500 |  | 0.2 | 0.327 |  | 0.3 | 0.292 |  | 0.3 | 0.292 |  | 0.3 | 0.292 |  | 0.3 | 0.292 |  | 0.1 | 0.376 |
|  | Ramet [R] |  | 6.5 | **0.005** |  | 8.5 | **0.002** |  | 8.2 | **0.002** |  | 7.3 | **0.003** |  | 4.2 | **0.020** |  | 3.4 | **0.033** |  | 2.4 | 0.061 |  | 2.3 | 0.065 |  | 2.9 | **0.044** |
|  | Cone (R) ^a^ |  | 2.3 | 0.065 |  | 1.6 | 0.103 |  | 0.6 | 0.219 |  | 0.7 | 0.201 |  | 1.2 | 0.137 |  | 1.3 | 0.127 |  | 2.1 | 0.074 |  | 2.1 | 0.074 |  | 1.4 | 0.118 |

1. Accounting for seed mass covariation

| Effects | |  | February | |  | March | |  | April | |  | May | |  | June | |  | July | |  | August | |  | September | |  | October | |
| --- | --- | --- | --- | --- | --- | --- | --- | --- | --- | --- | --- | --- | --- | --- | --- | --- | --- | --- | --- | --- | --- | --- | --- | --- | --- | --- | --- | --- |
|  |  | DF | *F*/χ2 | *P* value |  | *F*/χ2 | *P* value |  | *F*/χ2 | *P* value |  | *F*/χ2 | *P* value |  | *F*/χ2 | *P* value |  | *F*/χ2 | *P* value |  | *F*/χ2 | *P* value |  | *F*/χ2 | *P* value |  | *F*/χ2 | *P* value |
| *Fixed factors* | |  |  |  |  |  |  |  |  |  |  |  |  |  |  |  |  |  |  |  |  |  |  |  |  |  |  |  |
|  | Maternal environment [E] | 1, 9 | 0.0 | 0.877 |  | 0.1 | 0.767 |  | 0.2 | 0.684 |  | 0.3 | 0.602 |  | 0.3 | 0.589 |  | 0.1 | 0.739 |  | 0.3 | 0.572 |  | 0.6 | 0.452 |  | 0.8 | 0.387 |
|  | Block(E) ^a^ | 10, 30 | 1.0 | 0.481 |  | 0.8 | 0.666 |  | 0.7 | 0.747 |  | 0.7 | 0.753 |  | 0.7 | 0. 726 |  | 1.0 | 0.505 |  | 0.9 | 0.539 |  | 0.6 | 0.784 |  | 0.7 | 0.747 |
|  | Tray | 11, 1205 | 42.8 | **< 0.001** |  | 36.0 | **< 0.001** |  | 26.4 | **< 0.001** |  | 27.5 | **< 0.001** |  | 31.4 | **< 0.001** |  | 35.1 | **< 0.001** |  | 38.5 | **< 0.001** |  | 26.7 | **< 0.001** |  | 28.3 | **< 0.001** |
|  | Germination time | 1, 1205 | 55.5 | **< 0.001** |  | 56.7 | **< 0.001** |  | 58.8 | **< 0.001** |  | 63.1 | **< 0.001** |  | 52.6 | **< 0.001** |  | 36.7 | **< 0.001** |  | 23.5 | **< 0.001** |  | 20.0 | **<0 .001** |  | 16.1 | **< 0.001** |
|  | Seed mass | 1, 1205 | 98.3 | **< 0.001** |  | 103.7 | **< 0.001** |  | 99.9 | **< 0.001** |  | 97.7 | **< 0.001** |  | 90.0 | **< 0.001** |  | 75.8 | **< 0.001** |  | 58.7 | **< 0.001** |  | 37.7 | **< 0.001** |  | 30.8 | **< 0.001** |
|  |  |  |  |  |  |  |  |  |  |  |  |  |  |  |  |  |  |  |  |  |  |  |  |  |  |  |  |  |
| *Random factors* | |  |  |  |  |  |  |  |  |  |  |  |  |  |  |  |  |  |  |  |  |  |  |  |  |  |  |  |
|  | Maternal genotype [G] |  | 12.3 | **< 0.001** |  | 8.7 | **0.002** |  | 7.1 | **0.004** |  | 8.7 | **0.002** |  | 9.4 | **0.001** |  | 5.9 | **0.008** |  | 2.4 | 0.061 |  | 3.1 | **0.039** |  | 3.9 | **0.024** |
|  | G × E |  | 1.4 | 0.118 |  | 0.6 | 0.219 |  | 1.2 | 0.137 |  | 1.4 | 0.118 |  | 2.1 | 0.074 |  | 2.7 | 0.050 |  | 3.3 | **0.035** |  | 3.4 | **0.033** |  | 2.5 | 0.057 |
|  | Ramet [R] |  | 0.3 | 0.292 |  | 0.8 | 0.185 |  | 0.8 | 0.185 |  | 0.8 | 0.185 |  | 0.1 | 0.376 |  | 0.0 | 0.500 |  | 0.0 | 0.500 |  | 0.1 | 0.376 |  | 0.3 | 0.292 |
|  | Cone (R) ^a^ |  | 3.0 | **0.042** |  | 3.1 | **0.039** |  | 1.6 | 0.103 |  | 1.3 | 0.127 |  | 1.9 | 0.084 |  | 2.0 | 0.079 |  | 2.4 | 0.061 |  | 2.6 | 0.053 |  | 1.7 | 0.096 |

Seedlings were derived from 10 maternal genotypes clonally replicated in two contrasting maternal environments, one favourable and one unfavourable for pine growth and reproduction. Analyses excluding and including individual seed mass as a covariate are shown. Degrees of freedom (DF) and *F*-ratios of fixed factors, and associated χ^2^ of random factors are shown. Significance (*P* value) is indicated in bold (*P* < 0.05).

^a^ Block was nested within maternal environment and cone was nested within ramet.
